# Immediate Prosthesis Breast Reconstruction: A Comparison Between Ambulatory Surgery Versus Traditional Hospitalization Based on the Propensity Score Matching Method

**DOI:** 10.1007/s00266-022-03121-0

**Published:** 2022-10-06

**Authors:** Xiao Chen, Aoxiang Chen, Chaoqi Liu, Bin Zhang

**Affiliations:** The First Department of Breast Cancer, National Clinical Research Center for Cancer, Tianjin Medical University Cancer Institute and Hospital, North Huanhu West Road, Sports Institute, Hexi District, Tianjin, China

**Keywords:** Immediate prosthesis breast reconstruction, Breast-Q, Ambulatory surgery, Propensity score matching method, Complications

## Abstract

**Background:**

The positive benefits of immediate prosthesis breast reconstruction (IPBR) are incontrovertible. During the COVID-19 pandemic, health care resources became scarce. The implementation of outpatient immediate prosthesis breast reconstruction (OIPBR) can improve the efficiency of medical care and reduce viral exposure. Very few studies have focused on OIPBR and this study aimed to fill this gap by evaluating outcomes of OIPBR compared with traditional hospitalization IPBR (THIPBR) in terms of complications and quality of life.

**Material and Methods:**

The study enrolled patients undergoing IPBR at Tianjin Medical University Cancer Institute and Hospital between January 1, 2020, and September 30, 2021. Outcomes were defined as postoperative complications and quality of life before reconstruction and at 3-month follow-up. Quality of life was assessed by BREAST-Q questionnaire. Inverse probability of treatment weighting and propensity score matching (PSM) were applied to adjust for confounders.

**Results:**

A total of 135 patients were enrolled, including 110 with THIPBR and 25 with OIPBR. After matching, baseline characteristics were well balanced. Patients with OIPBR had lower rates of lymphedema on the surgery side (*p* = 0.041) and readmission (*p* = 0.040) than patients with THIPBR. No statistically significant differences in the quality of life metrics of psychosocial well-being, sexual well-being, satisfaction with breast and physical well-being of the chest were found between the two groups.

**Conclusion:**

OIPBR is a safe and efficient alternative to THIBPR during the COVID-19 pandemic. It is recommended when medical conditions allow to conserve medical resources. Accelerated technical training for the performance of OIPBR at the hospital level should be expedited.

**Level of Evidence III:**

This journal requires that authors assign a level of evidence to each article. For a full description of these Evidence-Based Medicine ratings, please refer to the Table of Contents or the online Instructions to Authors www.springer.com/00266.

## Introduction

Breast cancer is the most common malignancy in women, accounting for 16% of female cancers [1]. Survival rates for breast cancer patients have greatly improved in recent years but, where the integrity of the breast cannot be preserved, a negative impact on physical health and psychosocial well-being is often the result [2]. Thus, there has been a justified shift of focus toward the pursuit of breast integrity and aesthetics and a desirable treatment option is breast reconstruction. Breast implant reconstruction, which may be performed immediately or delayed, is the most commonly used method worldwide [3]. The current study focused on patients undergoing immediate prosthesis breast reconstruction (IPBR) by receiving surgical insertion of silicone prosthesis.

The COVID-19 epidemic has had a huge impact on the regular operation of healthcare facilities resulting in severe testing of the healthcare system [4]. While performing surgery, such as IPBR, the spread of COVID-19 must also be controlled. Other considerations which have led to scrutiny of ambulatory surgical procedures as an alternative to routine hospitalization of surgical patients include medical efficiency [5], costs to the healthcare service and economic burden to patients [6]. The performance of outpatient-based surgery, such as outpatient immediate prosthesis breast reconstruction (OIPBR), may serve to address many of these concerns [7]. Compared with OIPBR, traditional hospitalization IPBR (THIPBR) has a longer hospital stay, which increases the risk of virus infection to a certain extent and also increases the burden on the medical system, leading to a shortage of medical resources to a certain extent, especially during the epidemic. In terms of safety and quality of life, few studies have discussed in detail [8, 9]. Much of the existing research has been performed retrospectively, with the inherent risk of selection bias and confounding factors. Propensity score matching (PSM) is a statistical approach designed to eliminate selection bias by balancing the effect of confounding factors on the statistical results by the method of proportional matching [10]. The current analysis recruited and reviewed patients who underwent OIPBR at our hospital, applying the PSM method to adjust for confounding variables, and compared them with patients undergoing THIPBR. The aim of the study was to compare postoperative complications and quality of life between the two groups and provide reference material to ascertain whether OIPBR is suitable for further implementation during the COVID-19 epidemic.

## Materials and Methods

### Ethics Approval

The current study was conducted in accordance with the Declaration of Helsinki. Ethical approval was granted by the research ethics board of Tianjin Cancer Institute & Hospital. Informed consent was obtained from all individual participants included in the study.

### Studying Population and Anesthesia

**Fig. 1:**
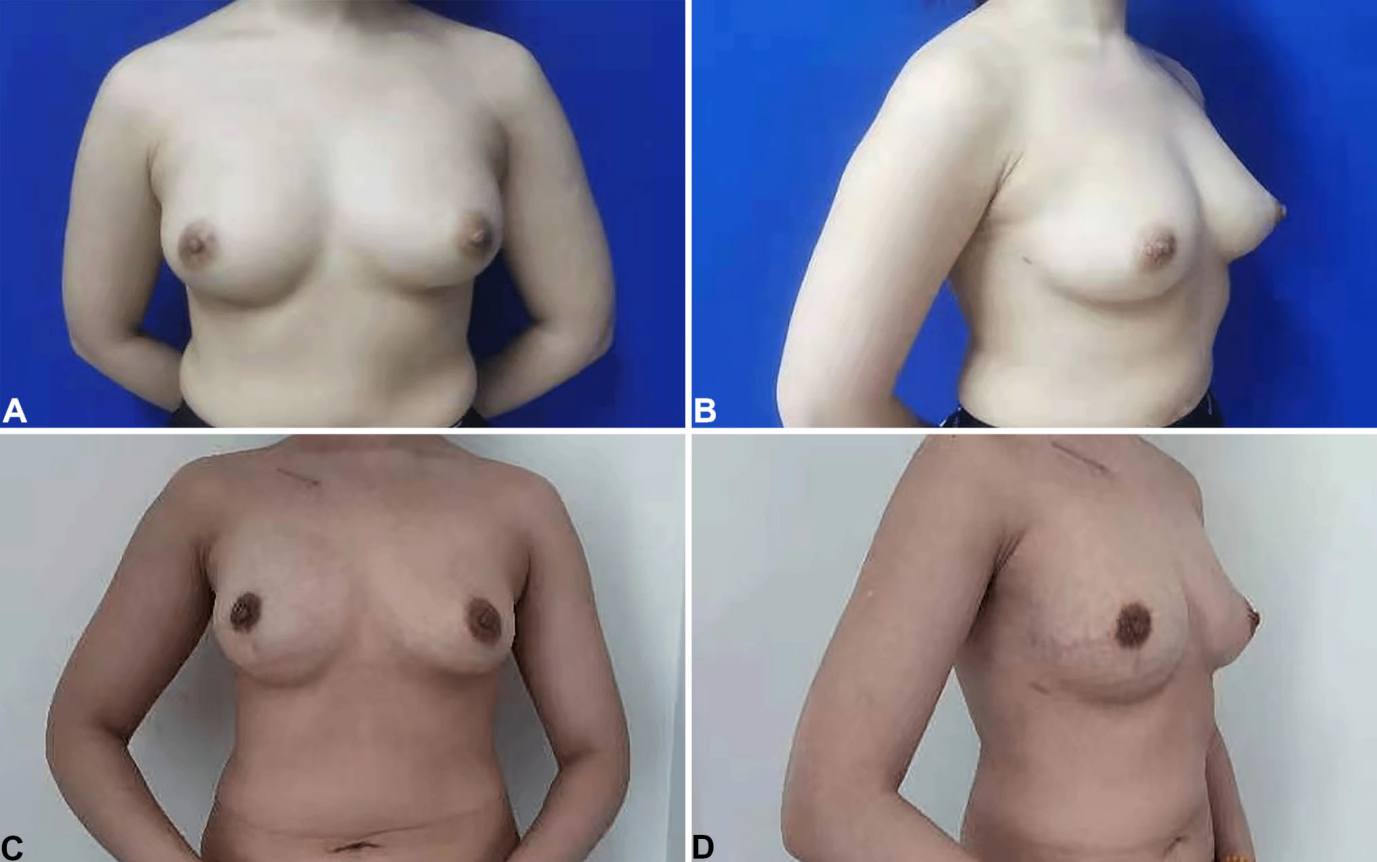
Outpatient immediate prosthesis breast reconstruction (OIPBR): **A**, **B** Before OIPBR. **C**, **D** Final result, 4 months after reconstruction

**Fig. 2:**
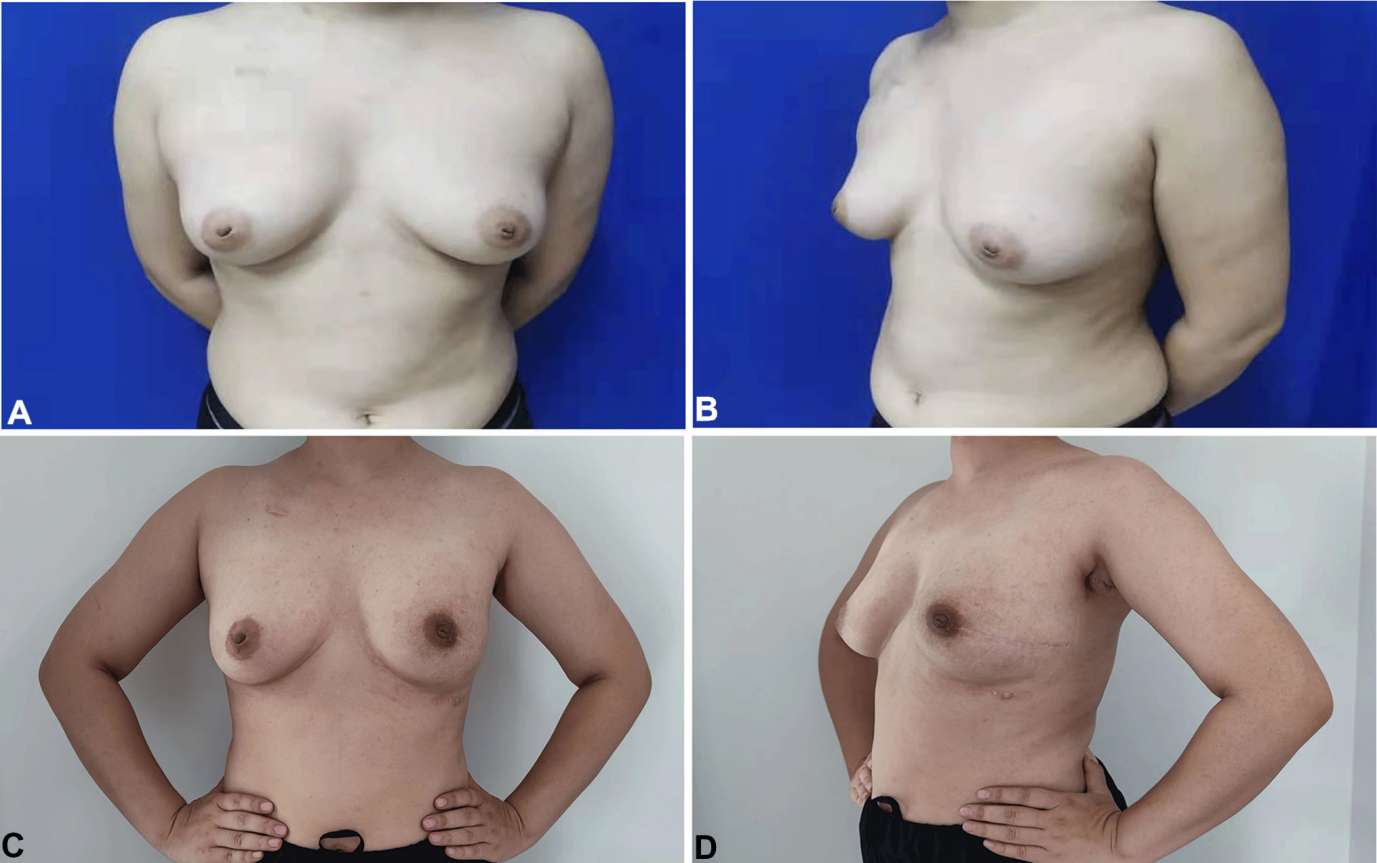
Traditional hospitalization immediate prosthesis breast reconstruction (THIPBR): **A**, **B** Before THIPBR. **C**, **D** Final result, 4 months after reconstruction

Patients who underwent IPBR performed by an associate chief surgeon of surgical oncology, together with 2 attending surgeons of surgical oncology, in Tianjin Medical University Cancer Institute and Hospital between January 1, 2020, and September 30, 2021, were enrolled. Ambulatory surgery is defined as an operation, excluding an office or outpatient operation, where the patient is discharged on the same working day. In terms of anesthesia, the anesthesiologist will assess the patient’s general condition and conduct preoperative visits to assess suitability for outpatient surgery. When deemed appropriate, doctors will prescribe drugs to prevent nausea and vomiting, and at the same time, achieve analgesia through various modes, and strengthen postoperative monitoring and care. Before discharge, the anesthesiologist will pay a return visit to the patient, which ensures the safety of OIPBR patients to a certain extent, and also accelerates the process of day surgery for patients. Patients were divided into two groups: OIPBR (Fig. 1) and THIPBR (Fig. 2), according to the definition of ambulatory surgery. Inclusion criteria were as follows: (1) Patients had a confirmed breast cancer diagnosis and were not candidates for breast conservation; (2) Patients had an American Society of Anesthesiologists (ASA) score ≤ II and body mass index (BMI) ≤ 35 kg/m^2^; (3) Patients had agreed to and had completed IPBR; (4) OIPBR patients had been accompanied by at least one other adult at discharge and lived close to the hospital. Patients were excluded who had opted for reconstruction on cancer recurrence following breast-conserving surgery.

Baseline variables were recorded for each patient as follows: age, BMI, tumor size, pathological tumor type, tumor grade, smoking status, education, neoadjuvant chemotherapy, postoperative chemotherapy/radiotherapy and immunohistochemical information, hormone receptor (HR), human epidermal growth factor receptor (HER2), Ki67. For patients receiving neoadjuvant chemotherapy, the analysis was performed based on immunohistochemical information prior to chemotherapy. HR was assigned a positive value when estrogen receptors and/or progesterone receptors were ≥ 1%. Tumors were considered to be in a proliferative state when Ki67 was ≥ 20%. The patient's hospital stay and surgery time were also recorded.

Study endpoints included postoperative complications, readmission, extubation time (time of removal of final drainage tube) and quality of life scores. Postoperative complications were as follows: (1) bleeding; (2) wound dehiscence; (3) flap ischemia; (4) surgical site infection; (5) lymphedema on the surgical side; (6) capsular contracture; (7) venous thromboembolism; (8) subcutaneous effusion; (9) cardiopulmonary complications.

The Breast-Q questionnaire is an instrument used to measure the quality of life and satisfaction of patients who have undergone breast surgery [11]. It considers aspects such as the patient’s satisfaction with breast, psychosocial well-being, physical well-being, sexual well-being, satisfaction with outcomes and satisfaction with care. An “A Score” is generated from the total score of the first four items in each category. Each patient completed the Breast-Q questionnaire prior to surgery (time point 1) and three months post-surgery (time point 2) to generate a score between 0 and 100, with higher scores indicating higher satisfaction and health-related quality of life [12].

### Statistical Analysis

Descriptive analysis of variables was used to summarize the data. Data showing a normal distribution are expressed as mean ± standard deviation (SDs) and non-normally distributed data as median and interquartile range (IQR). Percentage were used to describe continuous categorical variables. Differences between groups were analyzed by independent sample t-test, chi-square (*χ*^2^) test or nonparametric test and the 95% confidence interval (CI) calculated. The current study examined OIPBR performed as the result of a joint decision by the oncologic surgeon and the patient. To reduce bias in comparing non-randomized treatments, a propensity score was calculated for each subject. The propensity score was defined as the probability of treatment assignment conditional on the measured baseline covariates.

Baseline covariates of the current study were age, BMI, tumor size, pathological type, grade, smoking, education, neoadjuvant chemotherapy, postoperative chemotherapy/radiotherapy, immunohistochemical information (HR, HER2, Ki67) and quality of life score at time point 1. The propensity score was defined as the probability of accepting the OIPBR and was estimated using a logistic regression model. To control for confounders when the relationship between the two groups (THIPBR and OIPBR) and covariates was unknown, generalized boosted models or multivariate nonparametric regression techniques using inverse probability weighting (IPTW) were employed. IPTW weightings were estimated as the inverse of the patient’s estimated probability of belonging to the OIPBR. IPTW based on propensity scores was used to balance the distribution of baseline variables collated from the THIPBR and OIPBR groups. Baseline variables were considered to be balanced between the THIPBR and OIPBR groups if the absolute standardized mean difference was less than 0.1. All reported *p* values were two-sided and values of *p *< 0.05 were considered statistically significant. Analyses were performed using Excel version 2022, R software (version 4.1.2).

## Results

### Baseline Patient Information

Between January 1, 2020, and September 30, 2021, a total of 148 patients underwent IPBR in the hospital under consideration. One patient tested positive for the BRCA gene mutation, had a family history of breast cancer and was excluded. A further 8 patients refused to complete the questionnaire and four had recurrence of cancer necessitating reconstruction after breast-conserving surgery and were excluded. A total of 135 patients were thus eligible for inclusion. The mean time of hospitalization is 5.53 days. The mean time of the surgery in THIPBR group and OIPBR group was 144.02 minutes and 129.84 min, respectively.

Clinicopathological characteristics before and after IPTW adjustment are presented in Table 1. Before matching, there were 110 THIPBR patients with a median age of 43.54 years (SD = 7.46) and 25 OIPBR patients with a median age of 44.16 years (SD = 8.90). No significant differences between the two groups were present in terms of age, BMI, smoking, education, pathological type, neoadjuvant chemotherapy, postoperative chemotherapy/radiotherapy or Ki67 expression. However, significant differences in grade (*p* = 0.023), tumor size (*p* = 0.020) and HR/HER2 expression (*p* = 0.024) were found. Following PSM and IPTW adjustment, there was a good balance of baseline characteristics between the two groups.

**Table 1:** Patients’ clinicopathologic characteristics before and after PSM adjustment

	Before propensity score matching			After propensity score matching		
	THIPBR (*n* = 110)	OIPBR (*n* = 25)	*P*	THIPBR (*n* = 133.75)	OIPBR (*n* = 120.82)	*P*
Age (mean (SD))	43.54 (7.46)	44.16 (8.90)	0.717	43.76 (7.44)	45.08 (8.80)	0.550
Smoking (%)			1.000			0.598
Yes	6 (5.5)	1 (4.0)		6.90 (5.1)	3.50 (2.9)	
No	104 (94.5)	24(96.0)		126.85 (94.9)	117.32 (97.1)	
BMI (kg/m^2^) (%)			0.443			0.870
< 18.5	1 (0.9)	0 (0.0)		1.00 (0.7)	0.00 (0.0)	
18.5–23.9	58 (52.7)	9 (36.0)		66.60 (49.8)	54.00 (44.7)	
24.0–27.0	34 (30.9)	11 (44.0)		43.50 (32.5)	41.00 (34.0)	
> 27	17 (15.5)	5 (20.0)		22.60 (16.9)	25.80 (21.4)	
Education (%)			0.849			0.954
Primary school education	67 (60.9)	14 (56.0)		81.10 (60.6)	77.80 (64.4)	
Secondary school education	29 (26.4)	8 (32.0)		35.20 (26.3)	29.00 (24.0)	
Bachelor degree or above	14 (12.7)	3 (12.0)		17.50 (13.1)	14.00 (11.6)	
Pathological_type (%)			0.677			0.959
Ductal carcinoma	83 (75.5)	17 (68.0)		100.20 (74.9)	92.40 (76.5)	
Lobular carcinoma	5 (4.5)	2 (8.0)		7.30 (5.4)	7.40 (6.2)	
Other	22 (20.0)	6 (24.0)		26.20 (19.6)	20.90 (17.3)	
Grade (%)			0.023			0.148
I–II	77 (70.0)	13 (52.0)		91.80 (68.6)	93.40 (77.3)	
III–V	25 (22.7)	12 (48.0)		34.00 (25.4)	27.50 (22.7)	
Unknown	8 (7.3)	0 (0.0)		8.00 (6.0)	0.00 (0.0)	
Tumor_size (%)			0.02			0.641
≤ 20 mm	59 (53.6)	7 (28.0)		67.00 (50.1)	71.40 (59.1)	
> 20 and ≤ 50 mm	48 (43.6)	15 (60.0)		62.40 (46.6)	44.30 (36.7)	
> 50 mm	3 (2.7)	2 (8.0)		4.40 (3.3)	4.10 (3.4)	
Unknown	0 (0.0)	1 (4.0)		0.00 (0.0)	1.00 (0.8)	
Neoadjuvant chemotherapy (%)		0.692			0.715	
Yes	41 (37.3)	11 (44.0)		50.00 (37.4)	51.30 (42.4)	
No	69 (62.7)	14 (56.0)		83.75 (62.6)	69.52 (57.6)	
Postoperative chemotherapy (%)		0.228			0.515	
Yes	53 (48.2)	16 (64.0)		66.60 (49.8)	49.70 (41.1)	
No	57 (51.8)	9 (36.0)		67.15 (50.2)	71.12 (58.9)	
Postoperative radiotherapy (%)			0.276			0.239
Yes	37 (33.6)	5 (20.0)		41.00 (30.6)	21.20 (17.5)	
No	73 (66.4)	20 (80.0)		92.75 (69.4)	99.62 (82.5)	
HR_HER2 (%)			0.024			0.941
HR + /HER2−	71 (64.5)	10 (40.0)		81.40 (60.9)	75.10 (62.2)	
HR + /HER2+	19 (17.3)	4 (16.0)		24.60 (18.4)	23.70 (19.6)	
HR − /HER2+	9 (8.2)	7 (28.0)		13.80 (10.3)	13.60 (11.2)	
TN	11 (10.0)	4 (16.0)		13.90 (10.4)	8.40 (7.0)	
KI67 index (%)			0.400			0.805
< 20%	48 (43.6)	8 (32.0)		57.85 (43.2)	56.42 (46.7)	
≥ 20%	62 (56.4)	17 (68.0)		75.90 (56.8)	64.40 (53.3)	

*PSM* propensity score matching, *OIPBR* outpatient immediate prosthesis breast reconstruction, *THIPBR* traditional hospitalization immediate prosthesis breast reconstruction, *SD* standard deviation, *BMI* body mass index, *HR* hormone receptor, *HER2* human epidermal growth factor receptor.

### The Postoperative Complications of the Patients

Postoperative complications after PSM are given in Table 2. There was no significant difference in the incidence of bleeding between the OIPBR (3.40%) and the THIPBR (1.70%) groups (*p* = 0.536). THIPBR patients (4.20%) were more likely to have lymphedema on the surgical side than OIPBR (0.00%) patients (*p* = 0.041). The complication showing the highest incidence for both groups was capsular contracture (THIPBR: 29.90%; OIPBR: 14.40%), but differences were not significant (*p* = 0.229). No cardiopulmonary complications were seen for either group within three months of surgery. OIPBR patients had no complications such as wound dehiscence, flap ischemia, surgical site infection or venous thromboembolism, whereas 5 THIPBR patients were readmitted for surgical treatment for subcutaneous effusion, wound dehiscence or surgical site infection before PSM. After PSM, the two groups showed significant differences in readmission (*p* = 0.040). No difference in extubation time was found (*p* = 0.443).

**Table 2:** The postoperative complications of the patients after PSM

Characteristics	THIPBR (*n* = 133.75)	OIPBR (*n* = 120.82)	*P*
Bleeding (*n* (%))	4.50 (3.40)	2.10 (1.70)	0.536
Wound dehiscence (*n* (%))	3.40 (2.60)	0.00 (0.00)	0.109
Flap ischemia (*n* (%))	2.40 (1.80)	0.00 (0.00)	0.188
Surgical site infection (*n* (%))	2.20 (1.60)	0.00 (0.00)	0.188
Lymphedema of surgical side (*n* (%))	5.60 (4.20)	0.00 (0.00)	0.041
Capsular contracture (*n* (%))	40.00 (29.90)	17.40 (14.40)	0.229
Venous thromboembolism (*n* (%))	2.30 (1.70)	0.00 (0.00)	0.187
Subcutaneous effusion (*n* (%))	2.30 (1.70)	2.00 (1.60)	0.968
Cardiopulmonary complication (*n* (%))	0.00 (0.00)	0.00 (0.00)	NA
Readmission (*n* (%))	5.60 (4.20)	0.00 (0.00)	0.040
Extubation time (mean (SD)) (day)	34.20 (26.85)	31.45 (10.15)	0.443

*PSM* propensity score matching, *OIPBR* outpatient immediate prosthesis breast reconstruction, *THIPBR* traditional hospitalization immediate prosthesis breast reconstruction, *SD* standard deviation, *NA* not available.

### Quality of Life Improvements from Time Point 1 to 2

A trend toward higher psychosocial well-being scores was seen for the OIPBR patients (mean = 87.25 ± 14.02) relative to the THIPBR patients (mean = 81.60 ± 18.68) three months after surgery but this difference did not achieve statistical significance (*p* = 0.183; Table 3). No significant differences were found for sexual well-being (OIPBR: mean = 33.86 ± 36.56; THIPBR: mean = 48.25 ± 38.10; *p* = 0.144) satisfaction with breast (OIPBR: mean = 81.08 ± 9.23; THIPBR mean = 81.86 ± 14.74; *p* = 0.756), physical well-being of the chest (*p* = 0.391) or A score (*p* = 0.340). A scores showed a decreasing trend between the pre- and postoperative periods with the greatest decrease in sexual well-being. No inter-group differences were found for satisfaction with outcomes (*p* = 0.413) or satisfaction with care (*p* = 0.070). Thus, overall, the performance of OIPBR as opposed to THIPBR had no impact on patients’ perceived quality of life.

**Table 3:** Quality of life between Time point 1 and Time point 2

Score (mean (SD))	Time point	THIPBR (mean (SD))	OIPBR (mean (SD))	*P*
Psychosocial well-being	1	89.08 (11.66)	90.66 (13.29)	0.691
	2	81.60 (18.68)	87.25 (14.02)	0.183
Sexual well-being	1	63.95 (30.73)	62.73 (35.05)	0.885
	2	48.25 (38.10)	33.86 (36.56)	0.144
Satisfaction with breast	1	81.67 (15.03)	79.52 (16.88)	0.601
	2	81.86 (14.74)	81.08 (9.23)	0.756
Physical well-being chest	1	66.89 (12.19)	66.10 (15.00)	0.854
	2	59.01 (15.51)	56.19 (13.02)	0.391
A score	1	301.59 (44.00)	299.01 (35.25)	0.759
	2	270.71 (58.65)	258.38 (46.59)	0.340
Satisfaction with outcomes	–	98.97 (4.44)	96.98 (7.31)	0.413
Satisfaction with care	–	98.96 (4.47)	100.00 (0.00)	0.070

*SD* standard deviation, *OIPBR* outpatient immediate prosthesis breast reconstruction, *THIPBR* traditional hospitalization immediate prosthesis breast reconstruction, *Time point 1* time before surgery, *Time point 2* three-month post-surgery

## Discussion

One of the main outcomes of the present analysis is to provide further confirmation of the safety of OIPBR as an alternative to THIPBR. It is generally acknowledged that most IPBR-related complications are associated with age, personal medical history (smoking and overweight/obesity) and adjuvant therapy as risk factors. Once these factors had been matched for the subjects of the current study, no significant differences were seen between the two types of surgery [13, 14]. Evaluation of postoperative complications revealed that OIPBR patients had a significantly lower incidence of lymphedema on the surgical side (*p* = 0.041) and of readmission (*p* = 0.040) compared with THIPBR patients. Remaining postoperative complications and extubation time were not different. Quality of life assessments showed no significant differences in psychosocial well-being, sexual well-being, satisfaction with breast, physical well-being of the chest and A score between the two groups between the pre- and 3-month postoperative time points. Patients in the OIPBR group did, however, experience shorter hospital stays contributing to improved medical efficiency and financial savings and reducing COVID-19 transmission risk. Therefore, we believe that OIPBR surgery is a safe alternative to THIPBR when conditions permit.

It has previously been considered that IPBR was not an appropriate candidate for ambulatory surgery due to the potential for complications that are difficult to resolve [13]. However, the impact of the epidemic has exacerbated the scarcity of resources and capacity issues in cancer surgery [15]. Thus, OIPBR may represent a favorable way forward to reallocate medical resources while maximizing treatment efficiency for breast reconstruction patients. A large multicenter review summarizes the situation as one in which several studies have reported outcomes of IPBR complications and postoperative quality of life [16–18], but few have investigated ambulatory IPBR. A study completed by the Boston Division of Surgical Oncology and reported in Specht et al. showed that all 15 patients who underwent breast reconstruction at the center were discharged the same day with no postoperative complications or readmissions [8]. In the COVID-19 pandemic, Specht et al. provided us with a valuable lesson in OIPBR for breast cancer patients. Another study showed that patients who underwent IPBR and were discharged on the same day had a statistically insignificant difference in complications such as bleeding and infection (0.7%, 1.4%) compared with patients who underwent traditional inpatient surgery (0.0%, 0.8%) [19], which is consistent with our findings. In terms of readmission rates, the THIPBR group in our study (4.2%) was higher than the traditional hospitalization model group in that study (3.8%), but patients in OIPBR group (0.0%) were lower than those who underwent IPBR and were discharged on the same day of that study (8.0%). We consider that it may be related to adequate information to patients in OIPBR group. We adequately informed patients in both groups before the procedure and after discharge, but patients in OIPBR group had a short hospital stay, lacked professional medical care after discharge, and would be filled with anxiety about possible complications [20]. Our medical staff provides professional psychological support, using a patient-centered approach to encourage patients and families to ask questions and express their feelings which helps to alleviate the anxiety and fears of OIPBR patients and families about surgery and rapid discharge from the hospital [21]. Patients are also better prepared for home rehabilitation [22]. We also stipulate that patients undergoing ambulatory surgery should be accompanied by at least one adult and live close to the hospital at the time of discharge which also reduces patient and family anxiety. Given the extra pressures and anxieties contingent on the COVID-19 pandemic, ambulatory surgery reduces fears regarding SARSCov-2 infection [23]. Reducing patients’ anxieties across the spectrum has the effect of promoting wound healing and speeding up recovery from surgery [21].

The breast is an important sexual characteristic [24] and breast reconstruction improves its appearance and, thus, the patient’s quality of life [25]. Quality of life scores have been reported for patients with outpatient autologous breast reconstruction [26] but not yet for patients with OIPBR. The current study revealed no significant differences between OIPBR and THIPBR groups in any score either prior to surgery or at 3-month follow-up. Factors that influence satisfaction and quality of life after breast reconstruction include age, tumor stage, postoperative complications [14] and nicotine addiction [27], but some studies have also shown that postoperative implant complications are not associated with lower quality of life scores [28]. Nicotine addiction was strongly associated with breast satisfaction. The two groups of patients of the current study were matched for smoking status and no significant differences were found. Both OIPBR and THIPBR patients showed a non-significant increase in breast satisfaction after surgery compared to the preoperative period but patients in both groups developed capsular contracture at a higher rate than any other complication. Capsular contracture can lead to tenderness and altered shape of the reconstructed breast, as well as to numbness [29] and poor sexual experience leading to low sexual well-being scores [30]. Therefore, we also advocate support, help and understanding from partners of IPBR patients to optimize the sexual outcome after IPBR. Postoperative psychosocial well-being scores were not significantly higher for OIPBR patients than for THIPBR but online follow-up was established for all reconstructed patients, and proximity to the hospital and the ability to receive more timely and effective answers from doctors also helped to alleviate patients’ anxiety and nervousness [31, 32]. A prospective study has shown that IPBR leads to varying degrees of decline in chest and upper extremity function [33]. This was verified by the current conclusions but the majority of patients (92.3%) had recovered to their preoperative level at the final follow-up.

Several limitations of this study should be acknowledged. First, although patient characteristics were corrected by IPTW to minimize or eliminate bias, there may be some unknown confounders. Second, these are preliminary early results. Our follow-up period of 3 months was relatively short and late complications and patients’ quality of life require longer-term follow-up.

## Conclusion

The current study demonstrates that IPBR may be safely delivered via ambulatory surgery with no significant differences in quality of life scores or complications when compared with THIPBR. In response to the COVID-19 pandemic, implementation of OIPBR reduces patient-level viral exposure, facilitates rational use and allocation of healthcare resources and reduces the burden on the healthcare system. Not all hospitals have the capacity for OIPBR but mutual learning and training will allow it to become more widely available. We recommend the adoption of an evidence-based OIPBR system with patient follow-up network.

## Abbreviations

IPBR: Immediate prosthesis breast reconstruction
PSM: Propensity score matching
OIPBR: Outpatient immediate prosthesis breast reconstruction
THIPBR: Traditional hospitalization immediate prosthesis breast reconstruction
SD: Standard deviation
BMI: Body mass index
HR: Hormone receptor
HER2: Human epidermal growth factor receptor
